# Reversal of Direct Oral Anticoagulants (DOACs) for Critical Bleeding or Urgent Procedures

**DOI:** 10.3390/jcm14031013

**Published:** 2025-02-05

**Authors:** Mark Goldin, Nikolaos Tsaftaridis, Jack Jnani, Alex C. Spyropoulos

**Affiliations:** 1 Northwell, 2000 Marcus Ave., Suite 300, New Hyde Park, NY 11042-1069, USA; mgoldin@northwell.edu (M.G.); ntsaftaridis@northwell.edu (N.T.); jjnani@northwell.edu (J.J.); 2 Department of Medicine, Anticoagulation and Clinical Thrombosis Services, Northwell, New Hyde Park, NY 11042-1069, USA; 3 Institute of Health System Science, Feinstein Institutes for Medical Research, Manhasset, NY 11030, USA; 4 Donald and Barbara Zucker School of Medicine at Hofstra/Northwell, Hempstead, NY 11549, USA; 5 Department of Medicine, North Shore University Hospital, Manhasset, NY 11549, USA

**Keywords:** oral anticoagulants, bleeding, thrombotic disease, vitamin K

## Abstract

The advent of direct-acting oral anticoagulants (DOACs) has transformed the care of patients requiring prevention and treatment for thrombotic disease. Many randomized clinical trials have demonstrated the efficacy and safety of these agents and their comparative advantages over conventional anticoagulants such as vitamin K antagonists (VKAs). While historically clinicians and patients raised questions about the reversal of DOAC-associated bleeding, federal approval in recent years of targeted DOAC reversal agents, along with adjunctive modalities, has given clinicians reliable pharmacologic options. Yet, optimal reversal strategies for bleeding at specific anatomic locations and in specific clinical scenarios remains uncertain. We present here a narrative review of the literature on the reversal of DOAC-associated bleeding or for urgent procedures. The totality of the reversal literature synthesized here yields several clear conclusions: (1) targeted DOAC reversal with specific agents demonstrates superior efficacy for both bleeding and urgent surgical indications when compared to the use of non-specific agents, such as prothrombin complex concentrates (PCCs); (2) at the same time, high-quality data suggest potentially increased thrombotic risks, particularly for ischemic stroke, when using the specific targeted agent andexanet; (3) in all cases of life-threatening bleeding, timely reversal is of the essence; (4) in particular, there is growing consensus that DOAC-associated intracranial hemorrhage (ICH) should be reversed promptly, with a goal door-to-reversal time of 60 min; (5) future research will focus on optimizing clinical pathways for reversal to address “calls to action” from professional groups on this critical topic.

## 1. Introduction

There are few known clinicopathologic entities that rival thrombotic disease in global scope, depth and persistence of morbidity, and overall mortality. Thrombosis, which is the common mechanism of ischemic heart disease, ischemic stroke, and venous thromboembolism (VTE), has been implicated in 1 out of 4 deaths worldwide [1]. VTE—typically defined as deep vein thrombosis (DVT) and pulmonary embolism (PE)—and prevention of ischemic stroke in atrial fibrillation are common indications for extended anticoagulant treatment. While historically patients requiring anticoagulation relied heavily on vitamin K antagonist (VKA) therapy, in the past decade, direct-acting oral anticoagulants (DOACs) have largely become the anticoagulant therapy of choice and are recommended by major professional society guidelines on antithrombotic therapy [2,3].

DOACs offer distinct advantages over VKAs. They maintain predictable blood concentration without titration, are not substantially impacted by diet, and have reliable and relatively rapid elimination half-lives—all of which allow for simplified dosing. DOACs as a class reduce the risk of major and critical site bleeding—including intracranial hemorrhage (ICH) by about 50% compared to warfarin [4,5]. It has been shown that patients with poor warfarin time-in-therapeutic range can achieve better anticoagulant adherence when switching to DOACs [6]. Critically, better adherence, along with more predictable pharmacokinetic/pharmacodynamic profiles of DOACs, is associated with improved safety and efficacy compared with warfarin, as demonstrated by meta-analysis of individual patient-level data from pivotal DOAC trials [7].

Despite these safety advantages, major and life-threatening bleeding still occurs with DOACs, with annualized rates of 1.6% to 3.6% per year and ICH rates of ~0.3 to 0.5% per year, respectively, and urgent and emergent surgery needing anticoagulant reversal can occur in up to 2% of patients [8,9]. To address this need, several pharmacologic DOAC reversal options have emerged. In this narrative review, we will present a critical synthesis of published data with an eye toward resolving tradeoffs of available specific DOAC reversal agents, chiefly andexanet alfa and idarucizumab, compared to non-specific agents such as prothrombin complex concentrates (PCCs) and tranexamic acid. In recognition of the preponderance of data on central nervous system bleeding, principally ICH, we will also examine reversal clinical pathways and health system strategies to optimize hospital processes in these patients, especially by introducing the concepts of “Code ICH” and door-to-reversal time. Our review is organized by reversal indication (i.e., critical bleeding versus urgent surgery), agent, and type of evidence.

## 2. Methods

Literature searches of the MEDLINE database were undertaken during the initial drafting of this paper, looking for relevant articles published between 1986 and 2024 with the following keywords and MeSH terms: “Direct oral anticoagulant (DOAC)”, “Novel oral anticoagulant (NOAC)”, “Critical Bleeding”, “Acute Bleeding”, “Intracranial Bleeding”, “Gastrointestinal Bleeding”, “Factor Xa Inhibitor”, “Factor IIa Inhibitor”, “Reversal”, “Antidote”, “Surgical”, “Procedures”, and Operative”. Observational studies and randomized controlled trials on the topic of Factor Xa and FIIa Inhibitor reversal strategies prior to surgical procedures were included, as were meta-analyses and systematic reviews. Observational studies with fewer than 40 patients and case reports were excluded.

## 3. Results

### 3.1. Reversal of Critical Bleeding

A substantial majority of available data on reversal agents comes from studies of major or life-threatening bleeding. Much recent data relate to andexanet alfa (Table 1).

#### 3.1.1. Andexanet Alfa

Andexanet alfa is a reversal agent designed specifically to target factor Xa (FXa) inhibitors such as rivaroxaban and apixaban. Andexanet alfa acts as a decoy protein, binding FXa inhibitor molecules with high affinity similar to that of endogenous FXa, to neutralize their anticoagulant effect [40,41]. Since its phase 2 proof-of-concept and dose ranging studies over a decade ago [42,43], andexanet alfa has been evaluated in multiple studies for safety and efficacy, including large randomized controlled trials.

##### Randomized Controlled Trials

ANNEXA-I was the first randomized controlled trial comparing andexanet alfa to usual care (85% 4-factor PCC [4F-PCC]) in the management of acute ICH in patients on DOACs [10]. It included a safety analysis of 530 patients and efficacy analysis for 452 patients. It emphasized the challenging nature of finding the correct risk/benefit balance for the clinical setting given that it showed a 13.4% adjusted difference in the primary endpoint of hemostatic efficacy (hematoma expansion ≤ 35% at 12 h, increase in National Institutes of Health Stroke Scale/Score [NIHSS] of ≤7 points, and no receipt of rescue therapy between 3 and 12 h), as well as vastly superior median reduction in anti-FXa activity (94.5% versus 26.9%), but with a 4.6% absolute risk increase for thrombotic events, in particular ischemic stroke (6.5% versus 1.5%) (Table 1). Notably, the safety endpoint follow-up period for thrombotic events was 30 days, a short timeframe for assessing functional neurologic outcomes.

##### ANNEXA-4

While single-arm and open-label, the ANNEXA-4 clinical trial is the largest phase III study in the field of DOAC reversal and contributes some of the most important data in support of andexanet alfa. Indeed, ANNEXA-4 enabled provisional approval by the United States Food and Drug Administration (FDA) (Table 1). In ANNEXA-4, a multicenter, prospective study evaluating andexanet alfa in patients with acute major bleeding while on DOACs [11], 479 patients were enrolled. Bleeding was largely ICH (69%) and gastrointestinal (23%). Subjects received low-dose andexanet alfa if (1) their last FXa inhibitor dose was ≥8 h prior, or (2) if their last FXa inhibitor dose was <8 h prior and their FXa inhibitor dose was considered low (≤10 mg rivaroxaban, ≤5 mg apixaban, ≤40 mg enoxaparin, or <30 mg edoxaban). Excellent or good hemostasis was observed in 80% of patients at 12 h (95% Confidence Interval [CI] 75–84%). In the safety analysis, 50 patients (10%) had thrombotic events; all events occurred prior to resumption of oral anticoagulation. The median reduction in anti-FXa activity was 93% and 94% in patients treated with apixaban and rivaroxaban, respectively. Furthermore, it was observed that reductions in anti-FXa activity in patients over 75 years with ICH were associated with decreased mortality (*p* = 0.022).

Siepen and colleagues analyzed data from ANNEXA-4 along with the TICH-NOAC randomized controlled trial of tranexamic acid versus placebo with or without PCC for both groups [12]. This study showed that andexanet alfa was associated with decreased odds of hematoma expansion. The effect remained when comparing only to the PCC group of TICH-NOAC (adjusted odds ratio [OR] 0.21, 95% CI 0.08–0.53, *p* = 0.001). In TICH-NOAC, treatment with andexanet alfa was independently associated with decreased odds of hematoma expansion and the study was reassuring in terms of thromboembolic risk. Lower median modified Rankin Score (mRS) at follow-up was also seen with andexanet alfa, but the significance did not remain after sensitivity analysis. The authors concluded that the study was underpowered for mortality and functional outcomes.

Costa et al. compared andexanet alfa to 4F-PCC for ICH using data from ANNEXA-4 and a synthetic control arm with propensity score-overlap weighting [13]. In analyzing 202 patients (107 of which received andexanet alfa), andexanet alfa demonstrated both superior hemostatic effectiveness (OR 2.73, 95% CI 1.16–6.42) and lower 30-day mortality (OR 0.36, 95% CI 0.13–0.98) compared to 4F-PCC. Thrombotic events were rare and comparable between the two groups, though ANNEXA-4 only reported thrombotic events for 5 days (2 events with andexanet alfa versus 0 with 4F-PCC).

A third comparison of andexanet alfa to usual care for DOAC-related ICH combined data from ANNEXA-4 and the RETRACE-II registry [14]. Investigators used inverse probability of treatment weighing to adjust for baseline differences between 85 andexanet alfa-treated and 97 usual care (75.3% PCC)-treated patients. Most (85%) andexanet alfa-treated patients received a low dose. Average PCC dosing was near the low dose threshold. Hematoma expansion at 12 h for andexanet alfa and 36 h for usual care showed a relative risk of 0.44 (95% CI 0.22–0.88, *p* = 0.017). While there was a trend toward lower mortality in the andexanet alfa group (16.5% versus 20.6%, *p* = 0.48), the study was underpowered to detect significant differences in clinical outcomes, including mortality, thromboembolism, and functional outcomes (i.e., mRS).

Cohen and colleagues also compared andexanet alfa to PCC using data from ANNEXA-4 and the ORANGE observational study, via propensity score matching. Indications for reversal were predominantly ICH and gastrointestinal (GI) bleeding [15]. Andexanet alfa demonstrated significantly lower 30-day mortality compared to PCC in the overall cohort (14.6% versus 34.1%, adjusted RR 0.43, 95% CI 0.29–0.63). This mortality benefit was particularly pronounced in the ICH subgroup (15.3% versus 48.9%, RR 0.31, 95% CI 0.20–0.48) and similar trending but non-significant for GI bleeding (12.2% versus 25.0%, RR 0.49, 95% CI: 0.21–1.16). Important limitations included the inability to match for several highly predictive variables including Glasgow Coma Scale (GCS) score, hematoma volume, and expected survival.

##### Cohort Studies

In the largest multicenter cohort study (N = 4395) [16] comparing andexanet alfa versus 4F-PCC for DOAC-related major bleeding, regression and propensity score-weighted analyses showed lower in-hospital mortality (6.0% versus 10.6%, adjusted OR 0.50, 95% CI: 0.39–0.65, *p* < 0.01). This benefit was consistent across indications (ICH and GI bleeding). More than two-thirds (68.8%) of patients received low-dose andexanet alfa, and the median LOS was similar for both groups among survivors.

In a similar study comparing 265 patients receiving 4F-PCC with 59 patients receiving andexanet alfa, Sadek et al. did not find significant differences in mortality (25.4% versus 18.5%, adjusted OR 1.34, 95% CI 0.67–2.71), even in a subgroup with severe traumatic brain injury (28.6% versus 28.7%, OR 0.93, 95% CI 0.40–2.16) [17]. The study included only patients with an Abbreviated Injury Scale score > 2 that received andexanet alfa or 4F-PCC within 24 h of admission. The composite outcome of serious hospital complications included PE, stroke, DVT, reoperation, or ICU readmission and was also similar between groups (8.5% versus 7.9%, OR 1.01, 95% CI: 0.36–2.88). Hospital LOS was comparable, as was ICU LOS. There was a non-significant decrease in administration time for the andexanet alfa group (227 versus 385 min, adjusted OR 0.59, 95% CI: 0.16–2.16). According to the authors, this study was underpowered to detect non-inferiority. Key limitations included lack of data on exact DOAC timing, anticoagulation levels, and detailed neurological outcomes.

In a real-world, multicenter, retrospective analysis of andexanet alfa administration processes and clinical outcomes, data from 141 patients (83% ICH, 73.8% on apixaban) were analyzed [18]. The median time from presentation to andexanet alfa administration was 192.5 min (interquartile range [IQR] 108.0–337.0), with significantly longer times at tertiary hospitals (223.0 versus 130.0 min). There were three key components: ED presentation to diagnosis (72.5 min), diagnosis to order (35.5 min), and order to administration (53.0 min). The composite outcome of thromboembolism or major bleeding occurred in 22.7% of patients, with higher rates at tertiary centers (29.4% versus 5.1%). Overall mortality was 22.9%, with comparable rates across most indications except pre-surgical cases where no deaths occurred. The rate of thromboembolism was 10.6%.

In another multicenter retrospective study, Pham et al. found similar outcomes in terms of International Society on Thrombosis and Haemostasis (ISTH)-defined Excellent Hemostasis criteria (71.1% versus 70.7%, *p* = 1.0, adjusted *p* = 0.654) [19]. The median percentage change in hemorrhage volume at 12–24 h was also comparable (0% [−0.17–0.24] versus 0% [−0.021–0.29], *p* = 0.439), as were thromboembolic events (8.5% versus 9.7%, *p* = 1.0) and inpatient mortality (34.0% versus 21.0%, *p* = 0.134, adjusted *p* = 0.283). The order to administration time was substantially higher for andexanet alfa (70 versus 43 min) as were treatment costs (USD 23,602 versus USD 6692).

Sutton et al. retrospectively compared predominantly US Veteran Affairs patients treated with andexanet alfa (n = 85) versus 4F-PCC (n = 170) for DOAC- or enoxaparin-related major bleeding, finding significantly lower mortality rates with andexanet alfa both at 30 days (20.0% versus 32.4%, *p* = 0.039, unadjusted OR 0.52 [95% CI 0.28–0.96], propensity score-weighted hazard ratio (HR) 0.54 [95% CI 0.30–0.98]) and during hospitalization (10.6% versus 25.3%, *p* = 0.01, HR from cox proportional hazards model 0.34 [95% CI 0.16–0.74], propensity score-weighted HR 0.31 [95% CI 0.14–0.71]) [20]. GI bleeding was the predominant bleeding type (45.9% in the andexanet alfa versus 52.9% in the 4F-PCC group), with ICH the second most common (29.4% versus 28.8%), and finally other bleeding types (24.7% versus 18.2%). Blood product administration was similar between groups, as was LOS (11.3 versus 12 days, ratio 0.85 [95% CI 0.60–1.21]), with median ICU stays of 1 (IQR 0–4) versus 2 (IQR 0–5) days. Enoxaparin use was more common in the 4F-PCC group (<5% versus 37.7%). Andexanet alfa-treated patients had higher rates of discharge to home (57% versus 48.2%) and similar rates of discharge to nursing facilities (20% versus 15.9%) or transfer to other hospitals (9.4% versus 8.2%).

##### Case–Control Studies

In a single-center retrospective matched cohort analysis comparing andexanet alfa to 4F-PCC for DOAC-related ICH, 26 patients were matched to controls based on baseline ICH volume [21]. Excellent hemostasis (≤35% increase in ICH volume within 24 h) was similar (92.3% versus 88.5%, *p* = 1.000). Thrombotic events were not significantly different between groups (26.9% versus 11.5%, *p* = 0.159). Hospital LOS was similar as well (6.5 versus 4.5 days, *p* = 0.299). Most patients received low-dose therapy, while the median dose in the 4F-PCC group was close to 48.7 IU/kg. The authors also mention for this study as well that their analyses were underpowered for the outcomes studied.

In a single health system study across five hospitals in Michigan, Keinath et al. compared andexanet alfa (n = 170) to PCC (n = 170) for DOAC-associated bleeds (47% ICH, 37% GI), with the primary composite endpoint being deterioration-free discharge (hemostasis without mortality, need for packed red blood cells, hemoglobin decline after stabilization, unplanned interventions, or care escalation) [22]. This study was deemed as appropriately powered per the authors. There was no difference in the primary outcome (69.4% versus 66.5%, *p* = 0.646), mortality (16.5% versus 13.5%, *p* = 0.448), thrombotic events (5.3% versus 4.7%, *p* = 0.792), or hemostatic efficacy (81.8% versus 80.6%, *p* = 0.640). While 84% of andexanet alfa patients received low-dose therapy, 36% of high-dose administrations were inappropriate according to guidelines. Most PCC patients (87.6%) also received low-dose regimens (25 units/kg). No differences were found in hospital (5.9 versus 6.0, *p* = 0.383) and intensive care unit (ICU) (2.7 versus 2.1, *p* = 0.135) LOS. Expenses were significantly higher per deterioration-free discharge for andexanet alfa compared to PCC (USD 20,773.62 versus USD 5230.32, *p* < 0.001). Subgroup analyses of ICH (n = 159) and GI bleeding (n = 126) showed similar clinical outcomes but maintained the cost differential. Given that the data were not collected at the same time for both groups, there was the potential for additional bias in this study.

##### Additional Retrospective Studies

The literature also includes many smaller, single-center retrospective studies comparing andexanet alfa to 4F-PCC for DOAC-related bleeding, focused on ICH and to a lesser extent GI bleeding (Table 1). These demonstrated no significant differences in hemostatic efficacy, mortality, or thrombotic events between treatment groups [2,23,24,25]. One study reported that the median cost of care episode was higher with andexanet alfa [24] and a separate study found the median door-to-reversal times were about twice as long for GI bleeding as for ICH (1.8 h versus 3.6 h) [28].

##### Meta-Analyses

In a meta-analysis by Orso et al., andexanet alfa was compared to 4F-PCC for DOAC-related major bleeding [29]. It included randomized controlled trials, observational studies, propensity score matched studies, and interventional studies. There were 22 total studies, including 6 randomized controlled trial/propensity score matched studies and 16 retrospective studies of ICH and mixed bleeding. The primary outcome was short-term mortality and the secondary outcome was rate of thromboembolism. Randomized controlled trials and propensity score matched studies showed an RR of 0.71 (95% CI 0.37–1.34, I^2^ = 81%) for mortality and an RR of 1.74 (95% CI 1.09–2.77, I^2^ = 0%) for thromboembolism. The retrospective study analysis showed an RR of 0.82 (95% CI 0.63–1.07, I^2^ = 34.2%) for mortality and an RR of 1.18 (95% CI 0.86–1.62, I^2^ = 0%) for thromboembolism.

In another meta-analysis by Chaudhary et al., the authors compiled study-level results of 36 studies on adults with ICH providing data on safety (mortality and thromboembolic outcomes), as well as the proportion of patients in which anticoagulation was successfully reversed, for 4F-PCC, andexanet alfa, or idarucizumab [30]. In the sub-analysis of studies comparing andexanet alfa to 4F-PCC, eight studies were included. The authors concluded that no differences were found between the interventions in terms of mortality (RR 1.40 [95% CI 0.68–2.86]), thromboembolic outcomes (RR 0.89 [95% CI 0.36–2.21]), or the proportion of successful reversal (RR 0.95 [95% CI 0.85–1.06]).

#### 3.1.2. Idarucizumab

Idarucizumab is a humanized monoclonal antibody fragment that was designed as a specific antidote for the direct thrombin inhibitor dabigatran. Pre-clinical studies demonstrated that idarucizumab has an affinity for dabigatran approximately 350 times stronger than its affinity for thrombin [44].

##### REVERSE AD

The most robust—and FDA-enabling—data on idarucizumab comes from the REVERSE-AD clinical trial, a multicenter, prospective, open-label study evaluating the ability of idarucizumab to reverse dabigatran both in the setting of uncontrolled bleeding and prior to urgent surgery [31] (Table 1). Out of a total 503 patients analyzed, 301 (group A) presented with bleeding and 202 (group B) with a need for urgent surgery. Among group A patients, bleeding was predominantly gastrointestinal (45.5%) or ICH (32.6%). Idarucizumab achieved rapid and complete reversal of dabigatran (median reversal 100%), in all but three patients, while an important minority of patients (23.0%) had detectable rebound in dabigatran levels 12 h after idarucizumab administration. Among the 134 group A patients with clinical outcomes data, the median time to bleeding cessation was 2.5 h. Among group B patients who underwent surgery, 93.4% were adjudicated to have achieved normal periprocedural hemostasis. Thrombotic events occurred in 4.8% of patients within 30 days and 6.8% of patients within 90 days, similar to rates in previous studies [33]. Mortality at 30 days was relatively high—13.5% and 12.6% for groups A and B, respectively—for this very acutely ill, elderly, co-morbid population, in which many had suffered index trauma.

##### Retrospective Data

Singh analyzed data from a retrospective health system cohort comparing outcomes in patients treated with idarucizumab (n = 266) versus usual care (n = 1345) for dabigatran-associated major bleeding [32]. For patients with GI bleeding, idarucizumab showed no difference in terms of in-hospital mortality (5.9% versus 3.3%, adjusted OR 1.33, 95% CI 0.51–3.45), though it was associated with higher transfusion rates (60.8% versus 42.5%, *p* < 0.0001). There was higher in-hospital mortality in the idarucizumab group (11.6% versus 2.8%, *p* = 0.0011), though the authors note this was limited by sparse data and potential confounding of disease severity as the intervention group had worse prognosis at baseline. Thromboembolism rates were lower for both GI bleeding and ICH. LOS was significantly shorter with idarucizumab for ICH, but not for GI bleeding. No data were presented on what treatments the non-idarucizumab group received.

#### 3.1.3. Prothrombin Complex Concentrate

Prothrombin complex concentrate administration aims to replace vitamin K-dependent coagulation factors (II, VII, IX, and X) in DOAC-treated patients who are not factor depleted, without removing the anticoagulant effect of anti-FIIa or anti-FXa inhibitors [45]. The rate of hemostasis may be expected to be slower based on the rate of DOAC clearance.

##### Randomized Controlled Trial Data Analysis

In the RETRACE-II study [34], data from 131 patients with DOAC-related ICH were analyzed after excluding patients with primary intraventricular hemorrhage, those not on DOACs, and those without follow-up imaging/surgical evaluation (Table 1). Anti-Xa levels > 118 ng/mL on admission were associated with higher risk of hematoma expansion (56.2% versus 16.7%, RR = 3.375, 95% CI 1.245–9.115, *p* = 0.01), yet no association was found between PCC administration and reduced hematoma expansion (35.1% versus 35.1%, RR = 1.057, 95% CI 0.565–1.977, *p* = 0.863). This finding persisted regardless of PCC dosing or timing of administration. The study found no significant differences in 3-month mortality (no statistically significant difference between the groups) or functional outcomes (mRS 0–3: 31.1% versus 39.5%, *p* = 0.32). The study concluded that PCC showed no benefit in preventing hematoma expansion in this population.

##### Cohort Studies

In a multicenter prospective cohort study in Sweden, Majeed et al. evaluated PCC for DOAC-related major bleeding in 84 patients [35]. Bleeding was ICH in 70.2% of patients and GI in 15.5%. Based on ISTH hemostasis criteria, PCC was effective in 69.1% of cases. Patients most frequently received a low dose of 25 IU/kg. The 30-day mortality rate was 32%, with most deaths (74.1%) occurring in ICH patients. Thromboembolic complications were confirmed in only two patients post-PCC (2.4%). LOS was higher for patients with effective hemostasis (7 days [IQR 3.0–15.0]) compared to those that did not achieve hemostasis (4.5 days [IQR 2.0–7.0]).

In a prospective multicenter cohort study evaluating PCC for DOAC-associated major bleeding, 66 patients received a fixed dose of 2000 units PCC. Bleeding was predominantly ICH (55%) or GI (24%) [36]. The median time from bleeding onset to PCC administration was 8.6 h (IQR 4.8–18.1), with a door-to-PCC time of 5.4 h (IQR 3.3–7.8). Hemostatic effectiveness was rated good in 65% (95% CI 53–77), moderate in 20% (95% CI 10–30), and poor/none in 15% (95% CI 6–24) of patients. Reversal was effective in 68% of cases. Anticoagulation was resumed in 62% of patients at a median of 5 days (IQR 2–11.5).

Panos et al., conducted a large multicenter retrospective analysis of PCC use in 663 patients with DOAC-related ICH (intracerebral 45.1%; subdural 34.5%; subarachnoid 14.5%), 51% of which was traumatic [37]. Door-to-administration time was 2.6 h [IQR 1.5–4.3]. In-hospital mortality was 19%, with a rate of thromboembolism of 3.8%. Hospital and ICU LOS were 7.0 [IQR 3.7–12.0] and 2.8 [IQR 1.1–6.8] days, respectively. A second PCC dose was needed in 5.1% of cases.

In another recent retrospective multicenter effort, 125 patients were treated with either 4F-PCC (n = 64) or 3F-PCC (n = 61) [38]. The median door-to-needle time was 2–3 h. Agents demonstrated similar efficacy (82.8% versus 85.0%, adjusted OR 1.38, 95% CI 0.41–4.60, *p* = 0.81). In-hospital mortality (14.1% versus 14.8%, *p* > 0.999) and thromboembolism rates (4.7% versus 6.6%, *p* = 0.711) were also similar, as was hospital and ICU LOS (median 7.5 versus 8.0 days, and 3.0 versus 3.0 days, respectively). The median dose was moderate (50 UI/kg) in both groups. Repeat dosing was required only in the 4F-PCC cases at a low rate (3.1%). The PRBC transfusion rate was significantly higher in the 3F-PCC group (45.9% versus 18.8%, *p* = 0.002).

#### 3.1.4. Tranexamic Acid

The mechanism by which tranexamic acid exerts its antifibrinolytic activity is by competitively inhibiting the activation of plasminogen to plasmin [46]. Although initially used in hemophilia patients, it has seen expanded use in surgical and other bleeding scenarios. The TICH-NOAC randomized controlled trial compared tranexamic acid to placebo in patients with DOAC-associated ICH (N = 63) [39] (Table 1). There were no significant differences in the primary endpoint of hematoma expansion at 25 h (38% tranexamic acid versus 45% placebo, adjusted OR 0.63, 95% CI 0.22–1.82, *p* = 0.40). All thrombotic events occurred 2 weeks after ICH onset and exclusively in patients not restarted on anticoagulation. A pre-specified subgroup analysis suggested a potential benefit with earlier treatment (≤6 h from onset, P interaction = 0.024), hinting at the importance of early intervention. The tranexamic acid and placebo groups received 4F-PCC at similar rates, (22/32 (69%) versus 19/31 (61%), respectively). Tranexamic acid showed no clear benefit, though the study was underpowered.

### 3.2. Surgical Reversal

As noted, there are substantially less available data on reversal of DOACs for urgent surgery (Table 2). Large observational studies have suggested that patients who have preoperative DOAC assays are more likely to receive reversal therapy and have higher rates of bleeding and mortality [47], in addition to thromboembolism [48], likely all related to greater acuity at presentation. Godon et al. analyzed 478 patients on DOACs from the GIHP-NACO registry who required urgent procedures during 2013–2015, before DOAC-specific reversal agents were available [49]. Of all studied patients, 384 (80%) underwent surgical interventions, mostly orthopedic (216/384, 56%) and GI surgeries (75/384, 20%). Procedures were delayed in 194/455 (43%) of cases, and only 76/478 (16%) of patients received hemostatic agents. Only 62/478 (13%) of patients experienced excessive bleeding, and 30-day mortality was 28/478 (5.9%). While this study questioned the need for DOAC reversal before urgent procedures, its conclusions are contradicted by higher quality data on specific agents.

#### 3.2.1. Andexanet Alfa

In a small retrospective study, Bradshaw et al. assessed 44 patients on DOACs receiving andexanet alfa within 24 h of procedures including intracranial surgeries (N = 20, 45.5%), device placements (10, 22.7%), and arterial embolization procedures (9, 20.5%) [50]. Most (30/38, 78.9%) patients achieved excellent or good hemostasis within 24 h of andexanet alfa administration, with similar efficacy between intracranial (12/15, 80%) and extracranial events (18/23, 78.3%, *p* > 0.99). Thirty-day mortality reached 34.1% (15/44), and thromboembolic events occurred in 12/44 (27.3%) of cases, with a median time to a thrombotic event of 3.9 days (IQR 2.0–10.5]. The median door-to-reversal time was 2.6 h (IQR 1.2–5.5) and the time from reversal to procedure was 2.8 h (IQR 0.7–6.6). Hospital LOS was 11.5 days (IQR 6.0–19.0), and ICU LOS was 7.0 days (IQR 5.0–15.5). Prothrombin time and anti-Xa levels decreased significantly, as did blood product usage after reversal for extracranial bleeding (45.8% pre versus 12.5% post, *p* = 0.03), but not for intracranial bleeding (15% pre versus 45% post, *p* = 0.08).

#### 3.2.2. Idarucizumab

In urgent surgical settings, idarucizumab has shown rapid reversal of dabigatran. As described earlier, the REVERSE-AD study included an urgent surgery arm of 202 patients (study group B) (Table 2) [31]. In a sub-analysis of the REVERSE-AD study, Levy et al. evaluated 202 patients who underwent abdominal (n = 49, 24%), orthopedic (n = 45, 22%), vascular (n = 34, 17%), neurologic (n = 8, 4%), and other (n = 66, 32.7%) procedures [51]. Idarucizumab yielded rapid reversal of dabigatran with normal hemostasis in over 91% of patients within a median time of 1.2–1.9 h, except in neurosurgery cases where hemostasis required a median of 3.3 h (range 0.4–130.4). Complete reversal was achieved in >95% of patients based on direct thrombin time and in 90% based on aPTT. Thromboembolic events occurred in 10 patients (5%) and 30-day mortality was 12.6%.

#### 3.2.3. Prothrombin Complex Concentrate and Factor Eight Inhibitor Bypass Activity

PCC and activated PCC (aPCC) have also been evaluated as reversal strategies before surgery. Shaw et al. (N = 82) reported “Normal” hemostasis in 84% (21/25) of operative cases after aPCC and substantial reductions in INR and aPTT [52]. This study recorded a relatively low thromboembolic event rate (6.1%), suggesting aPCC’s feasibility as a possible alternative in cases where bleeding control is essential, yet the risk of thromboembolism remains a concern. Thirty-day all-cause mortality was 31.7% (26/82 patients) and fatal bleeding events occurred in 17 (20.7%) patients.

Similarly, Barzilai et al. examined 62 elderly patients on apixaban or rivaroxaban over the age of 80 years, primarily undergoing abdominal surgery (61%) [53]. After PCC, only 5% of patients experienced significant bleeding and only 3% thromboembolic complications. Thirty-day mortality (21%) was largely due to comorbid conditions like sepsis rather than bleeding or thromboembolism.

Engelbart et al. conducted a case series of 42 patients treated with factor eight inhibitor bypass activity (FEIBA), a complex consisting mainly of non-activated factors II, IX, and X, as well as activated factor VII [54]. Patients presented with life-threatening hemorrhage or urgent need for a procedure and received a median dose of 30 units/kg (IQR 25–37). Successful hemostasis was achieved in 86% of patients (36/42). Mortality (29%), hemorrhage progression (10%), and thrombotic event rates (10%) were similar to rates in other aPCC studies. The median door-to-needle time was 136 min (IQR 92–181). Thromboembolic complications occurred in four patients after FEIBA administration (median time-to-event 4 days).

Emigh et al. analyzed the use of FXa reversal agents in 606 elderly (median age 78 years) trauma patients (81% with falls) on DOACs undergoing urgent surgeries [55]. Overall, only 75 patients (12%) were reversed, and of those, 60 (80%) received 4F-PCC, nine (12%) received idarucizumab, five (7%) received FEIBA, and one (1%) received andexanet alfa. Patients who received a drug-specific reversal agent (i.e., idarucizumab or andexanet alfa) had significantly shorter hospital and ICU LOS, and fewer ventilator days, but marginally higher mortality versus those reversed with non-specific agents (OR 14.9, 95% CI 1.4–155.6, *p* = 0.04).

Considering the observational nature and mostly small size of available studies on DOAC reversal for urgent surgery, as well as the fact that many mixed anticoagulant type studies only include a small proportion of subjects on DOACs, there is a need for higher quality data. The ongoing ANNEXA-RS randomized controlled trial (NCT05926349) aims to enroll and randomize 800 patients to evaluate andexanet alfa versus usual care within 15 h of DOAC exposure. The trial’s results are expected to provide guidance on the use of andexanet alfa across various surgical procedures, with secondary outcomes including 30-day mortality, thromboembolic complications, and LOS.

## 4. Critical Synthesis, Discussion, Conclusions

While DOACs have superior net clinical benefit among available oral anticoagulants, these potent drugs still pose important safety risks, chiefly major bleeding. An effective antidote must rapidly reverse DOAC effects while minimizing pro-thrombotic effects. As presented in this review, the reversal literature predominantly focuses on the indication of critical bleeding, as opposed to urgent surgery, and moreover, studies are largely oriented on central nervous system bleeding such as ICH. There is now a body of high-quality randomized trial data, and taken with substantial observational data, there are clear advantages in using specific reversal agents as compared with non-specific agents.

Since the approval of andexanet alfa, and especially in recent years, there has been much debate about the need for randomized controlled trial data. In this context, much of the discussion over the results of ANNEXA-I [10] has “missed the forest for trees”. This high-quality randomized controlled trial demonstrated clear efficacy of reduction in hematoma expansion of ICH in a large population, but a concerning safety signal, in the form of increased risk of ischemic stroke, which has confused interpretation. However, the incidence of thrombotic events was similar to that observed in ANNEXA-4. More importantly, standard calculation using ANNEXA-I efficacy and safety outcomes reveals a number needed to treat (1/absolute risk reduction) of ~7.5 versus a number needed to harm (1/absolute risk increase) of nearly 22; in other words, at the cost of one thrombotic event, about three hematoma expansion events can be averted [10]. It is also worth considering that ANNEXA-I was not designed and was underpowered to capture mortality or longer-term functional outcomes, so the significance of increased adjudicated stroke outcomes on patient quality of life is uncertain. The authors of ANNEXA-I readily conceded that their study was not designed—nor could it feasibly be designed—to measure such longer-term outcomes, and they accordingly noted the difficulty of assessing net clinical benefit. This transparent shortcoming should not deter andexanet alfa use, both because of the intrinsic advantages of andexanet alfa and the lack of high-quality data for other reversal agents. Of note, post hoc data from Annexa-I suggest an optimal time of symptom onset for ICH of 6 h or less and an index hematoma volume of approximately >5 mL to <35 mL to optimize reversal outcomes compared to usual medical care [56]. Patient-level observational comparisons using ANNEXA-4 data [12,13,14,15] for central nervous system bleeding have clearly shown an advantage in hemostatic efficacy for andexanet alfa in the context of reduction in anti-FXa activity when compared with PCC, as well as trends toward lower short-term (30-day) mortality. Of particular importance, two recent, large-scale, observational comparisons of andexanet alfa and 4F-PCC, across thousands of hospitalizations at hundreds of U.S. centers, have shown an association with markedly reduced in-hospital mortality (50–60%) for andexanet alfa in multivariate analyses [16,57]. Moreover, these reductions were consistent across bleeding types, highlighting broad clinical applicability.

Even lower-level evidence has shown at least the equivalence of andexanet alfa with PCC on additional outcomes, including hospital and ICU LOS, need for administration of PRBCs, and other measures of in-hospital deterioration [22,25,26,27,28].

These results were consistent across dosing regimens and varying indications (ICH versus GI versus mixed bleeding). Despite many studies being underpowered, the trends toward improved functional outcomes with andexanet alfa, particularly mRS, are encouraging; while the potential for an increase in stroke is real, possibly from a mechanism of sequestering TFPI [58], these studies suggest that increased follow-up may reveal improvement in neurologic functional outcomes. Taken with meta-analytic data showing potential short-term mortality benefits with andexanet alfa versus PCC—albeit amid a backdrop of high study heterogeneity—there is ample evidence to support andexanet alfa as first-line therapy in ICH. These data credibly extend to other major bleeding.

Idarucizumab has been studied in very ill populations requiring reversal for both uncontrolled bleeding and urgent surgery. In the REVERSE-AD trial [31], the drug demonstrated a ready ability to completely reverse dabigatran within 4 h based on varied laboratory assessments. Moreover, out of 503 subjects, only nine (1.8%) received repeat doses of idarucizumab, only three of whom were experiencing recurrent bleeding. Sub-analyses and additional retrospective data affirm an unequivocal net clinical benefit.

Data focused on non-specific agents are less robust. There are currently no randomized controlled trial data demonstrating the efficacy of PCC in ICH. Even in small cohort studies, PCC has rarely demonstrated hemostatic efficacy for more than 2/3 of patients and without assessments of DOAC anticoagulant activity before reversal [35,36,37]. There are also no data to support tranexamic acid use in ICH, and generally this remains a niche agent used in surgical patients not specifically on DOACs.

**Figure 1:**
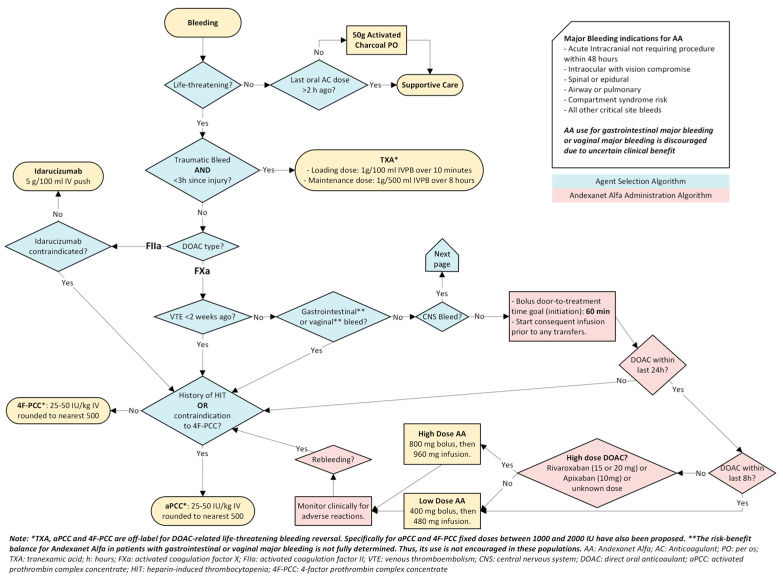
Oral Anticoagulant Reversal for Patients with Major Bleeding: Agent selection and Andexanet Alfa Administration Algorithms.

**Figure 2:**
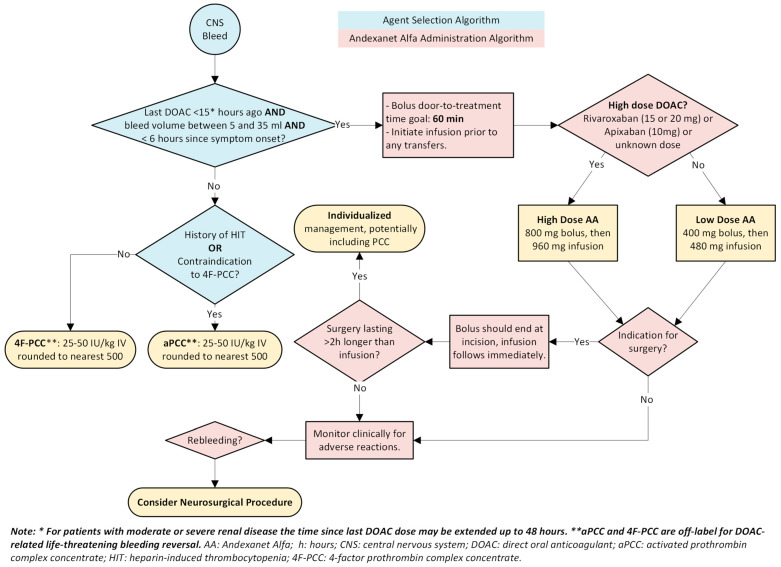
Oral Anticoagulant Reversal for Patients with Major Bleeding: Agent selection and Andexanet Alfa Administration Algorithms, Continued: Section for Central Nervous System Bleeding.

The totality of data reveals an undeniable conclusion: specific agents such as andexanet alfa and idarucizumab are supported as an effective DOAC reversal agent in the most potentially catastrophic and debilitating bleeds, particularly ICH. Non-specific agents are not supported as viable alternatives currently. A proposed algorithm for DOAC-associated critical site or life-threatening bleeding based on the best available evidence is shown in Figure 1 and Figure 2.

Efforts would be better spent on developing clinical pathways for the efficient administration of specific reversal agents. Observational studies to date have demonstrated that door-to-reversal with andexanet alfa times are commonly 2.5 to 4 h [18,50,59], substantially longer than the 1 h window suggested for improved survival [60]. This goal has been affirmed in a recent “call to action” for standardizing processes, such as with multidisciplinary “Code ICH” teams at major centers [61].

The surgical reversal data are generally lower quality and are challenging to compare due to small, vastly different study populations. The high efficacy of andexanet alfa is counterbalanced by relatively high mortality and thromboembolism in the context of very high-risk surgeries—largely neurologic and intravascular procedures [50]. PCC and aPCC, in contrast, have shown lower thromboembolic complications, but in lower-risk scenarios, such as abdominal procedures including percutaneous cholecystostomy and ERCP [52,53]. Indeed, when related concentrate products like FEIBA have been used in higher-risk surgical settings, mortality has been correspondingly higher [54,55]. Accordingly, health professionals responsible for system policies would wisely circumscribe reversal recommendations to major bleeding, pending additional surgical data.

## Figures and Tables

**Table 1:** Summary of clinical studies evaluating anticoagulation reversal for acute bleeding indications, including Andexanet Alfa, PCC, Idarucizumab, and Tranexamic Acid. Abbreviations: ICH, Intracranial Hemorrhage; GI, Gastrointestinal; FXa, Factor Xa; RCT, Randomized Controlled Trial; HE, Hemostatic Efficacy; PCC, Prothrombin Complex Concentrate; DOAC, Direct Oral Anticoagulant; OR, Odds Ratio; aOR, adjusted Odds Ratio; RR, Relative Risk; CI, Confidence Interval; LOS, Length of Stay; DVT, Deep Vein Thrombosis; PE, Pulmonary Embolism; TIA, Transient Ischemic Attack; MI, Myocardial Infarction; VTE, Venous Thromboembolism; mRS, Modified Rankin Scale; GCS, Glasgow Coma Scale; TXA, Tranexamic Acid; CNS, Central Nervous System; aPCC, Activated Prothrombin Complex Concentrate; ISTH, International Society on Thrombosis and Haemostasis.

Study	Design	Intervention vs. Comparator	Indication	Primary Outcome (Int vs. Comp)	Mortality (Int vs. Comp)	Thrombo-embolism (Int vs. Comp)	Hemostasis (Int vs. Comp)	Additional Details
**Andexanet Alfa**								
**Trials**								
Connolly, 2024 (N = 452 for efficacy, 530 for safety) [10]	RCT	Andexanet vs. Usual Care	ICH	HE ≤ 35% at 12 h, increase in NIHSS of ≤7 points and no receipt of rescue therapy between 3 and 12 h	27.8% vs. 25.5%, Increase per 100 pts 2.5 (95% CI: −5.0 to 10.0), *p* = 0.51	10.3% vs. 2.8%, Increase per 100 pts 4.6 (95% CI: 0.1 to 9.2), *p* = 0.048	67.0% vs. 53.1%, Adjusted Difference 13.4% (95% CI: 4.6 to 22.2), *p* = 0.003	
Milling, 2023 (N = 342 for hemostatic efficacy) [11]	Multicenter, prospective single-group cohort	Andexanet vs. N/A	Mixed (ICH 69.1%, GI 22.8%, Other 8.1%)	Anti-FXa activity reduction: Apixaban: 93% reduction from 146.9 to 10.0 ng/mL (95% CI, 94–93); Rivaroxaban: 94% reduction from 214.6 to 10.8 ng/mL (95% CI, 95–93); Edoxaban: 71% reduction from 121.1 to 24.4 ng/mL (95% CI, 82–65); Enoxaparin: 75% reduction from 0.48 to 0.11 IU/mL (95% CI, 79–67); Hemostatic efficacy: 274 of 342 evaluable patients achieved excellent or good hemostasis (80% [95% CI, 75–84])	15.7% at 30 days (75/479); ICH mortality 16.9% (56/331), GI bleeding 11.9% (13/109), other major bleeding 15.4% (6/39)	50 patients (10.4%) had ≥1 thrombotic event within 30 days: 15 DVT, 7 PE, 22 ischemic strokes, 3 TIA, 10 MI. 19 events occurred within 6 days, 31 events at 6–30 days	80% excellent/good at 12 h (274/342 evaluable patients). By FXa inhibitor: Rivaroxaban 81% (95% CI, 73–87), Apixaban 79% (95% CI, 72–85), Enoxaparin 88% (95% CI, 62–98), Edoxaban 79% (95% CI, 59–92)	One severe infusion reaction reported. No neutralizing antibodies to factor X, FXa, or andexanet alfa developed. Of 323 (67.4%) patients who received anticoagulation during follow-up, none of the 130 who restarted oral anticoagulation had thrombotic events after restart. Study conducted at 85 centers across North America (26), Europe (49), and Japan (10). Median endogenous thrombin potential was within normal range by end of andexanet alfa bolus through 24 h for all FXa inhibitors.
**Annexa-4-related trials**								
Siepen, 2024 (N = 243) [12]	Prospective individual data analysis	Andexanet vs. TXA or placebo +/− PCC	ICH	HE at 12–24 h: 24 (14%) vs. 26 (41%), *p* < 0.001, Crude OR: 0.24 (95% CI: 0.12–0.46), *p* < 0.001. After adjustment: OR: 0.33 (95% CI: 0.13–0.80), *p* = 0.015	30-day mortality 36/180 (2050 vs. 25/63 (40%) *p* = 0.002, crude OR: 0.28 (95% CI: 0.12–0.66), *p* = 0.004, adjusted OR 0.46 (985% CI: 0.18–1.23), *p* = 0.123	Overall thromboembolism at 30 days 20/180 (11%) vs. 6/64 (10%), Crude OR: 1.19 (95% CI: 0.45–3.10, *p* = 0.726)	HE: 24/280 (41%) vs. 26/63 (14%), *p* < 0.001 OR 0.24 (95% CI: 0.12–0.46), adjusted OR: 0.33 (95% CI: 0.13–0.80, *p* = 0.015	Absolute hematoma volume change: −7.12 mL (95% CI: −11.41 to −2.83), *p* = 0.0013
Costa, 2022 (N = 202) [13]	Prospective individual data analysis	Andexanet vs. 4F-PCC	ICH	Hemostatic effectiveness 85.8% vs. 68.1% 4F-PCC (OR 2.73, 95% CI 1.16–6.42)	7.9% vs. 19.6% (30d), OR 0.36 (95% CI: 0.13–0.98)	1% vs. 0% no odds ratio or *p* value reported	68.1% vs. 85.8%, OR 2.73 (95% CI: 1.16–6.42) no *p* value reported	Most patients received low-dose andexanet (96.6%) and 79.3% of patients received 4F-PCC (25 units/kg) Most patients had an indication of Afib for DOACs. And 61% of ICHs were trauma-related
Huttner, 2022 (N = 182) [14]	Case–control	Andexanet vs. Usual Care	ICH	HE ≥ 35% at 12 h for AA or 36 h for usual care	16.5% vs. 20.6%, *p* = 0.48	12.9% vs. 10.1%, *p* = 0.632	HE ≥ 35%: 14% vs. 36%, RR 0.40 (95% CI: 0.20–0.78), *p* = 0.005	Adjusted Hematoma volume reduction: −7.12 mL (95% CI: −11.41 to −2.83), *p* = 0.0013.
Cohen, 2022 (N = 410) [15]	Case–control with propensity score matching	Andexanet vs. PCC	Mixed 282 ICH, 137 GI, 48 other)	30-day mortality: 14.6% vs. 34.1%, adjusted RR 0.43, 95% CI: 0.29–0.63	14.6% vs. 34.1%, Adjusted RR 0.43 (95% CI: 0.29–0.63); ICH subgroup mortality: 15.3% vs. 48.9%, RR 0.31 (95% CI: 0.20–0.48); GI bleed subgroup mortality: 12.2% vs. 25.0%, RR 0.49 (95% CI: 0.21–1.16)	N/A	N/A	Adjusted 30-day mortality: 14.60% (95% CI: 10.72–18.47) vs. 34.09% (95% CI: 23.99–44.19); Study limitations included inability to match for certain prognostic variables like GCS score and hematoma volume
**Cohort Studies**								
Dobesh, 2023 (N = 4395) [16]	Cohort	Andexanet vs. 4F-PCC	Mixed	Mortality	6.0% vs. 10.6%, adjusted OR 0.50 (95% CI: 0.39–0.65), *p* < 0.01	N/A	N/A	ICH mortality: 12.6% vs. 23.3%, adjusted OR 0.55 (95% CI: 0.39–0.76), *p* < 0.01; GI bleed mortality: 2.5% vs. 4.3%, adjusted OR 0.49 (95% CI: 0.29–0.81), *p* = 0.01; LOS: Mean 7.1 vs. 6.9 days; Median 6.0 days (IQR 4.0, 8.0) for both groups;
Sadek, 2024 (N = 324) [17]	Cohort	Andexanet vs. 4F-PCC	ICH (traumatic)	Mortality/hospice at discharge: 15 (25.4%) vs. 49 (18.5%), adjusted OR 1.34, 95% CI: 0.67–2.71	25.4% vs. 18.5%, OR 1.34 (95% CI: 0.67–2.71)	N/A	N/A	ICU LOS: median 2 vs. 2 days, OR 0.98, 95% CI: 0.84–1.14; ISS > 25 47.5% vs. 26.1%, *p* = 0.002; Minutes to operation: 227 vs. 385 adjusted OR 0.59, 95% CI: 0.16–2.16; LOS: median 4 vs. 4 days, adjusted OR 0.93, 95% CI: 0.83–1.05
Goldin, 2024 (N = 141) [18]	Retrospective	Andexanet, no comparator	Mixed (ICH 83.0%, Other CNS 2.8%, GI 2.1%, Other critical 9.9%, Pre-surgery 5.0%)	Presentation to andexanet time: 192.5 min (IQR 108.0–337.0)	22.9% overall (ICH 22.4%, Other CNS 25.0%, GI 33.3%, Other critical 25.0%, Pre-surgery 0%)	VTE or ATE: 10.6% overall	Major bleeding: 12.0% overall	Composite of post-andexanet VTE, ATE, or major bleeding: 22.7% overall. 17% were transfers. 73.8% were on apixaban. Overall: 192.5 min (IQR 108.0–337.0); Tertiary centers: 223.0 min (IQR 142.0–358.0); Non-tertiary centers: 130.0 min (IQR 87.0–253.0); ED to diagnosis: 72.5 min (IQR 39.0–137.5); Diagnosis to order: 35.5 min (IQR 0–96.5); Order to administration: 53.0 min (IQR 38.5–78.5) Tertiary hospitals had longer process times across the board.
Pham, 2022 (N = 109) [19]	Cohort	Andexanet vs. 4F-PCC	ICH	Excellent hemostasis: (71.1% vs. 70.7%, *p* = 1.0)	16 [34.0%] vs. 13 [21.0%], *p* = 0.134, Adjusted *p* = 0.283	4 [8.5%] vs. 6 [9.7%], *p* = 1	71.1% vs. 70.7%, *p* = 1.0	Adjusted *p*-value for mortality: 0.283; ICU LOS (3.0 vs. 3.0 days), diff 0.0 d (95% CI: −2.2 to 1.2); LOS: (6.7 vs. 5.1 days), diff 1.6 d (95% CI: −3.1 to 3.0)
Sutton, 2023 (N = 255) [20]	Cohort	Andexanet vs. 4F-PCC	Non-ICH	30-day Mortality: 20.0% vs. 32.4%, *p* = 0.039, unadjusted OR 0.52 (95% CI: 0.28–0.96)/HR from cox proportional hazards model 0.54 (95% CI: 0.30–0.98)/Propensity score-weighted HR: 0.54 (95% CI: 0.30–0.98); (in-hospital) 10.6% vs. 25.3%, *p* = 0.01, unadjusted OR 0.52 (0.28–0.96)/HR from cox proportional hazards model 0.34 (95% CI: 0.16–0.74)/Propensity score-weighted HR: 0.31 (95% CI: 0.14–0.71);	Mortality: 20.0% vs. 32.4% (30 d), *p* = 0.039, OR 0.52 (95% CI: 0.28–0.96)/HR from cox proportional hazards model 0.54 (95% CI: 0.30–0.98); (in-hospital) 10.6% vs. 25.3%, *p* = 0.01, OR 0.52 (0.28–0.96)/HR from cox proportional hazards model 0.34 (95% CI: 0.16–0.74)	N/A	N/A	mean ICU LOS: 4.2 vs. 3.9 days; median ICU LOS: 1 (IQR: 0–4) vs. 2 (IQR: 0–5) days; Discharge disposition: Home 57% s 48.2%, VA/Community nursing home: 20% vs. 15.9%, transfer to other hospital: 9.4% vs. 8.2%; LOS: 11.3 vs. 12 days, ratio 0.85 (95% CI: 0.60–1.21);
**Case–control studies**								
Parsels, 2022 (N = 52) [21]	Case–control	Andexanet vs. 4F-PCC	ICH	Good/excellent hemostasis within 24 hof administration: 92.3% vs. 88.5%, *p* = 1.000	No data	New event within 14 days: 26.9% vs. 11.5%, *p* = 0.159	92.3% vs. 88.5%, *p* = 1.000	LOS: 6.5 vs. 4.5 days, *p* = 0.299
Keinath, 2023 (N = 340) [22]	Case–control	Andexanet vs. PCC or aPCC	Mixed (ICH 47%, GI 37%, Other 16%)	Deterioration-free discharge: (69.4% vs. 66.5%, *p* = 0.646)	16.5% vs. 13.5%, *p* = 0.448	5.3% vs. 4.7%, *p* = 0.792	81.8% vs. 80.6%, *p* = 0.640	Primary outcome (deterioration-free discharge) was composite of: mortality, hemostasis within 24 h, level of care elevation, need for additional PRBCs, hemoglobin drop after normalization, and unplanned interventions. ICU LOS: 2.7 vs. 2.1 days, *p* = 0.135; Cost per deterioration-free discharge: USD 20,773.62 vs. USD 5230.32, *p* < 0.001; Discharge to home: 30.6% vs. 31.8%, *p* = 0.411; Discharge to home 49.2% vs. 46.3%, *p* = 0.746; LOS: 5.9 (IQR not reported) vs. 6.0 days, *p* = 0.383
**Additional retrospective studies**								
Lipski, 2022 (N = 70) [23]	Cohort	Andexanet vs. 4F-PCC	ICH (traumatic 68%, spontaneous 32%)	Excellent or good hemostatic efficacy at 12 h post-reversal: 75% vs. 66.7%, *p* = 0.62	28-day: 39.1% vs. 40.4%, *p* = 0.92	28-day: 21.7% vs. 17.0%, *p* = 0.63	Excellent/good at 12 h: 75% vs. 66.7%, *p* = 0.62	ICU LOS: Median 3.5 vs. 3 days, *p* = 0.75; Only evaluable patients were included in hemostasis analysis (AA = 12, 4F-PCC = 21); Higher baseline ICH scores in AA group (median 3 vs. 2, *p* = 0.03); Similar rates of surgical intervention after reversal (30.4% vs. 17.0%, *p* = 0.20); There was a significant difference in DOAC distribution between the groups; Time to restart anticoagulation was 149.9 ± 160.8 h for 4F-PCC group and 51.2 ± 28.3 h for the AA group, *p* = 0.345; 4F-PCC: All patients received fixed 50 units/kg dose; AA dosing: Low dose (400 mg bolus + 4 mg/min): 73.9%, High dose (800 mg bolus + 8 mg/min): 26.1%; Mortality rates higher (40%) than previously published literature (26–38%), attributed to tertiary center. High rate of traumatic ICH (~68% in both groups). Nearly half had multicompartmental hemorrhage (46.8% vs. 52.2%). Baseline ICH volumes: 18.3 mL (AA) vs. 15.7 mL (4F-PCC) (*p* = 0.25); LOS Median 7 vs. 7 days, *p* = 0.61
Irizarry-Gatell, 2024 (N = 89) [24]	Retrospective	Andexanet vs. 4F-PCC	ICH	30-day all-cause mortality: 52% vs. 35%, *p* = 0.14	30-day: 52% vs. 35%, *p* = 0.14; in-hospital: 22% vs. 26%	13% vs. 26%, *p* = 0.17	Radiographic stability: 57% vs. 58%, *p* = 0.93; Objective improvement in ICH volume: 10% vs. 8%, *p* = 0.18; Progression of ICH: 24% vs. 23%, *p* = 0.74	ICU LOS: Median 4d [IQR 2–7] vs. 3d [IQR 0–7], *p* = 0.5; Cost: USD15,000 [IQR USD 15,000-USD 27,000] vs. USD 11,650.90 [IQR USD 8567-USD 14,149]; Progression of ICH: 24% vs. 23% (*p* = 0.74); LOS: Median: 7d [IQR 6–12] vs. 6d [IQR 3–12], *p* = 0.66
Koo, 2024 (N = 183) [25]	Cohort	Andexanet vs. 4F-PCC	Mixed	Hemostatic efficacy Excellent or Good: 75% vs. 69.7%, Difference: 5.3% (95% CI: −7.9% to 18.5%), *p* = 0.43	11.9% vs. 20.2%, Difference: 8.3% (95% CI: −2.2% to 18.8%), *p* = 0.122	7.1% vs. 7.1%, OR 1.0 (95% CI: 0.34–2.96), *p* = 0.985	75% vs. 69.7%, Difference: 5.3% (95% CI: −7.9% to 18.5%), *p* = 0.43; Corrected % decrease in Hb: Overall 11.9% vs. 15.7%, *p* = 0.004; ICH only: 10% vs. 12.4%, *p* = 0.076; GIB only: 21.9% vs. 31.37%, *p* = 0.562;	Time to administration: 43.5 vs. 46 min, *p* = 0.621; ICU LOS: 2 vs. 2 days, *p* = 0.015; Required surgical intervention: 16.7% vs. 16.2%, *p* = 0.927; LOS: 7 vs. 6 days, *p* = 0.439
Vestal, 2022 (N = 56) [26]	Retrospective case series	Andexanet vs. 4F-PCC	ICH	N/A	14.3% vs. 37.1% (deceased) 14.3% vs. 2.9% (hospice)—no further metrics provided	14.3% vs. 31.4%	64.7% vs. 54.8%	ICU LOS: 3.78 [2.54–6.69] vs. 2.29 [1.37–5.83] days; Hospital LOS: 7.75 [4.64–15.87] vs. 5.02 [2.72–8.56] days; Time to administration: 2.67 [1.75–4.13] vs. 1.73 [1.21–3.55] hours; Prophylactic anticoagulation resumption: median 59.4 [47.6–69.9] hours after administration (both groups combined); Systemic anticoagulation restart time: Andexanet: 26.1 [13.8–35.0] days, 4F-PCC: 14.7 [9.3–24.0] days; Hospital: 7.75 [4.64–15.87] vs. 5.02 [2.72–8.56] days ICU: 3.78 [2.54–6.69] vs. 2.29 [1.37–5.83] days
Schmidt, 2022 (N = 85) [27]	Retrospective	Andexanet vs. 4F-PCC	Non-ICH	N/A	18.1% vs. 17.3%, *p* = 0.918	18% vs. 3.8%, *p* = 0.027	84.8% vs. 76.9%, *p* = 0.373	ICU LOS: 2.5 vs. 2.5 days, *p* = 0.516; 6.2 vs. 5.4 days, *p* = 0.577
Singer, 2023 (N = 100) [28]	Cohort (Retrospective Pilot)	Andexanet vs. 4F-PCC	ICH (50), GIB (50)	Hemostatic efficacy: 88% vs. 76%, OR 2.01 (95% CI: 0.67–6.06), *p* = 0.8; Rebleeding rates: 2% vs. 4%, OR 3.30 (95% CI: 0.59–18.52)	8% vs. 9%, *p* = 0.25	14% vs. 16%, *p* = 0.8	88% vs. 76%, OR 2.01 (95% CI: 0.67–6.06), *p* = 0.8; Rebleeding rates: 2% vs. 4%, OR 3.30 (95% CI: 0.59–18.52)	Door-to-needle time for ICH: median 1.8 h (andexanet) vs. 1.7 h (4F-PCC); Door-to-needle time for GIB: median 3.6 h vs. 3.3 h; For GIB bleeding only: hemostatic efficacy (excellent): 64% vs. 44%; Survival to discharge: 76% vs. 88% (*p* = 0.46).
**Meta-analyses**								
Orso, 2024 [29]	Meta-analysis	Andexanet vs. 4F-PCC	Mixed	All-cause mortality RR 0.84 (95% CI: 0.69–1.01);	RCTs and PSMs: RR 0.71 (95% CI 0.37–1.34), I^2^ = 81%	RCTs and PSMs: RR 1.74 (95% CI 1.09–2.77), I^2^ = 0%	N/A	Significant publication bias in retrospective studies (Egger’s test *p* = 0.03)
Chaudhary, 2022 (N = 1832) [30]	Review	Andexanet vs. 4F-PCC and Idarucizumab	Mixed	All-cause mortality and Thromboembolic outcomes	AA: 24% (95% CI, 16–34%) vs. 4F-PCC: 26% (95% CI, 20–32%); Retrospective study RR 1.40 (95% CI: 0.68–2.86);	AA: 14% (95% CI, 10–19%) vs. 4F-PCC: 8% (95% CI, 5–12%); Retrospective RR 0.89 (95% CI: 0.36–2.21);	AA: 75% (95% CI, 67–81%) vs. 4F-PCC: 77% (95% CI, 72–82%); Retrospective study RR: 0.95 (95% CI: 0.85–1.06)	Subanalysis (4F-PCC vs. AA): Anticoagulation reversal RR 0.95 (95% CI, 0.85–1.06)
**Idarucizumab**								
Pollack, 2017 (N = 503) [31]	RCT (multicenter, prospective, single-cohort)	Idarucizumab, no comparator	Mixed (Group A: uncontrolled bleeding [45.5% GI, 32.6% ICH]; Group B: urgent surgery)	Maximum percentage reversal of dabigatran anticoagulant effect: 100% (95% CI: 100–100)	30-day: 13.5% Group A, 12.6% Group B; 90-day: 18.8% Group A, 18.9% Group B	30-day: 4.8% (24/503); 90-day: 6.8% (34/503)	Group A: median time to bleeding cessation 2.5 h (95% CI: 2.2–3.9) in evaluable patients; Group B: 93.4% normal, 5.1% mildly abnormal, 1.5% moderately abnormal periprocedural hemostasis	Median time to procedure 1.6 h in Group B; Time from last dabigatran dose median 15.6 h; Reversal was rapid and maintained for 24 h in most patients; Single 5 g dose sufficient in 98% of patients; Anti-idarucizumab antibodies in 5.6% of patients
Singh, 2019 (N = 1611) [32]	Cohort	Idarucizumab, usual care	Mixed	N/A	GI: 5.9% vs. 3.3% (aOR 1.33, 95% CI 0.51–3.45), ICH: 11.6% vs. 2.8%, *p* = 0.0011	GI: 1.3% vs. 4.2%, *p* = 0.0825; ICH: 0.9% vs. 10.1%, *p* = 0.0018	N/A	Transfusion aOR 2.0 (95% CI 0.64–6.30), 30-day readmission aOR 0.8 (95% CI 0.38–1.68), Cost: USD 26,240 vs. USD 21,201, *p* = 0.1864; LOS: GI: 7.8 vs. 8.8 days (IRR 0.92, *p* = 0.125) ICH: 8.1 vs. 11.4 days (IRR 0.82, *p* = 0.03)
**Retrospective data**								
Sarode, 2013 (N = 202) [33]	Phase IIIb multicenter open-label, non-inferiority RCT	4F-PCC vs. Plasma	Mixed—major bleeding while on VKA therapy	Co-primary endpoints: Hemostatic efficacy at 24 h: 72.4% 4F-PCC vs. 65.4% plasma (difference 7.1%, 95% CI: −5.8 to 19.9), demonstrating noninferiority; INR correction (≤1.3) at 0.5 h after infusion: 62.2% 4F-PCC vs. 9.6% plasma (difference 52.6%, 95% CI: 39.4 to 65.9);		7.8% (8/103) 4F-PCC vs. 6.4% (7/109) plasma Treatment-related: 3.9% (4/103) vs. 2.8% (3/109)	Hemostasis—Excellent: 44.9% vs. 43.3%; Good: 27.6% vs. 22.1%; Poor/none: 27.6% vs. 34.6%; Overall excellent/good: 72.4% vs. 65.4%;	Median infusion time: 17 min 4F-PCC vs. 148 min plasma; Median infusion volume: 99.4 mL 4F-PCC vs. 813.5 mL plasma; Fluid overload events: 4.9% 4F-PCC vs. 12.8% plasma; Treatment-related adverse events: 9.7% 4F-PCC vs. 21.1% plasma; Baseline median INR: 3.90 (1.8–20.0) 4F-PCC vs. 3.60 (1.9–38.9) plasma; Most patients received vitamin K in addition to study treatment
**PCC**								
**RCT Data analysis**								
Gerner, 2018 (N = 131) [34]	Cohort	PCC vs. no PCC	ICH	Hematoma enlargement > 33%: 35.1% vs. 35.1%, RR = 1.057 (95% CI: 0.565–1.977), *p* = 0.863	19.9% at discharge, 29.5% at 3 m (overall cohort)—no significant difference and no specifics in terms of comparison mentioned	N/A	Hematoma enlargement > 33%: 35.1% vs. 35.1%, RR = 1.057 (95% CI: 0.565–1.977), *p* = 0.863	Systolic BP < 160 mmHg at 4 h: RR 0.598 (95% CI: 0.365–0.978) for hematoma enlargement, mRS 0–3 at 3 m: 31.1% vs. 39.5%, *p* = 0.32
**Cohort studies**								
Majeed, 2017 (N = 84) [35]	Cohort	PCC, no comparator	Mixed (70. 2% ICH; 15.5% GI Bleeding)	Effective hemostasis per ISTH criteria: overall, 69.1% (58/84) patients; ICH only, 72.9% (43/59);	32% (27/84) at 30 days; 18% (15/84) died within first week;	2 confirmed ischemic strokes, 1 suspected PE	Effective in 72.9% (43/59)	Median PCC dose: 2000 IU (IQR 1500–2000) or 26.7 IU/kg (IQR 21.4–29.9); LOS: Median 7.0 days (IQR 3.0–15.0) for those with effective hemostasis; Median 4.5 days (IQR 2.0–7.0) for ineffective
Schulman, 2018 (N = 66) [36]	Cohort	PCC, no comparator	Mixed	Effective hemostasis	14% (9 deaths)	8% (5 events within 30 days)	65% good (95% CI: 53–77), 20% moderate (95% CI: 10–30), 15% poor/none (95% CI: 6–24)	ICU LOS: Median 0 days (IQR 0–6); Post hoc ISTH hemostatic criteria ICH (n = 36): Good: 67% Moderate: 17% Poor/None: 17%; GI (n = 16): Good: 69 Moderate: 12% Poor/None: 19%; Timing: onset-to-PCC 8.6 h (IQR 4.8–18.1), door-to-PCC 5.4 h (IQR 3.3–7.8); AC resumed in 62% at median 5 d (IQR 2–11.5); Mean age 76.9 y; AF main indication (82%); ICH types: 18 intracerebral, 7 subdural, 4 SAH, 7 combinations; Major TE: 3 strokes, 1 Peripheral arterial embolism, 1 VTE; 8/9 deaths in ICH patients (22% ICH mortality); LOS: 16 days median (IQR 5.3–30)
Panos, 2020 (N = 663) [37]	Cohort	PCC, no comparator	ICH (51% Traumatic; Intracerebral (45.1%), Subdural (34.5%), Subarachnoid (14.5%))	Hemostatic efficacy (433/663 patients were evaluable for this); 81.8% (95% CI, 77.9–85.2) excellent/good	19.0% in-hospital	3.8% in-hospital	81.8% (95% CI, 77.9–85.2) excellent/good	ICU LOS: Median 2.8 days (IQR 1.1–6.8); Median time to PCC administration: 2.6 h (IQR 1.5–4.3); Anticoagulation reinitiation in 5.9% of patients, Median time to reinitiation: 8.2 days [IQR 5.2–13.9] from admission; Median initial dose of 4F-PCC (43.8 u/kg [IQR 25.6–49.8]); Median initial dose of aPCC 26.7 u/kg [IQR 23.8–48.3]; 34 patients (5.1%) received a second PCC dose; LOS: Median 7.0 days (IQR 3.7–12.0)
Hays, 2024 (N = 125) [38]	Cohort	4F-PCC vs. 3F-PCC	Mixed	Effective hemostasis: 82.8% (53) vs. 85% (51), adjusted OR 1.38 (0.41–4.60) *p* = 0.81	In-hospital mortality 14.1% (9) vs. 14.8% (9), *p* > 0.999	4.7% (3) vs. 6.6% (4), *p* = 0.711		ICU LOS: 3 (IQR 4.8) vs.. 3 (IQR 6); LOS median 7.5 d, IQR 10 vs. 8 d, IQR 7
**Tranexamic Acid**								
Polymeris, 2023 (N = 63) [39]	RCT	TXA vs. Placebo	ICH	HE ≥ 33% or ≥6 mL: 38% vs. 45%, OR 0.63 (95% CI: 0.22–1.82), *p* = 0.40	47% vs. 42%, OR 1.07 (95% CI: 0.37–3.04), *p* = 0.91	13% vs. 6%, OR 1.86 (95% CI: 0.37–9.50), *p* = 0.45	HE: 38% vs. 45%, OR 0.63 (95% CI: 0.22–1.82), *p* = 0.40, Symptomatic HE: 28% vs. 29%, OR 0.86 (95% CI: 0.28–2.66), *p* = 0.79	mRS 0–4 at 90 d: 44% vs. 52%, RR 0.81 (95% CI: 0.29–2.27), *p* = 0.69 Terminated early (planned N = 109) Subgroup analysis suggested benefit with treatment ≤ 6 h. 69% of the TXA group and 61% of the placebo group received 4F-PCC

**Table 2:** Summary of studies evaluating reversal strategies, clinical outcomes, and hemostatic efficacy in patients receiving anticoagulant therapy who underwent urgent or emergent surgical procedures. Abbreviations: DOACs = Direct Oral Anticoagulants; PCC = Prothrombin Complex Concentrate; FFP = Fresh Frozen Plasma; aPCC = Activated Prothrombin Complex Concentrate; FEIBA = Factor Eight Inhibitor Bypass Activity; TXA = Tranexamic Acid; PT = Prothrombin Time; aPTT = Activated Partial Thromboplastin Time; INR = International Normalized Ratio; HR = Hazard Ratio; RR = Relative Risk; OR = Odds Ratio; CI = Confidence Interval; EVD = External Ventricular Drain; ERCP = Endoscopic Retrograde Cholangiopancreatography; AIS = Abbreviated Injury Scale; ISS = Injury Severity Score; LOS = Length of Stay.

Study	Design	Indication and Procedure	Intervention | Comparator	Mortality (Int vs. Comp)	Thromboembolism (Int vs. Comp)	Hemostasis (Int vs. Comp)	Bleeding as Complication	Additional Details
Yoo, 2020 (N = 1984) [48]	Retrospective	Hip fracture surgery, ≥60 years old, on any anticoagulant (97.9% Warfarin, 2.1% DOACs)	Vitamin K, PCC, FFP, idarucizumab|No reversal	30-day mortality 7.8% vs. 6.0%, unadjusted HR 1.30 (95% CI 0.82–2.07), Adjusted HR 1.00 (95% CI 0.62–1.60)	Thromboembolism: unadjusted RR 2.70, 95% CI 1.18–6.04; Adjusted RR 3.14, 95% CI 1.34–7.40)	Hemoglobin drop of at least 3 g/dL: 20.7% vs. 24.0%; RR 0.86 (95% CI 0.69–1.07), not statistically significant.	The study did not detect a significant difference in major bleeding (defined as hemoglobin drop ≥3 g/dL) between the reversal group and non-reversal group (RR 0.86, 95% CI, 0.69–1.07).	Higher delirium rates in reversal group (8.6% vs. 4.9%, RR 1.77, 95% CI: 1.08–2.89); Surgery within 24 h associated with lower mortality risk before adjustment; Only 3.6% on concurrent antiplatelet therapy; Reversal group had higher admission INR on average (2.6 vs. 2.0, *p* < 0.001); Time to surgery longer in reversal group (47.1 vs. 37.0 h, *p* < 0.05). LOS: 6.4 vs. 5.8 days (adjusted mean difference 0.08, 95% CI −0.55–0.71)
Stretton, 2023 (N = 1065) [47]	Multicenter Retrospective	Surgery in patients on DOACs requiring pre- and post-op hemoglobin measurement. Spinal (11.9%), Orthopedics (15.4%), Neurosurgery (19.4%)	Preoperative DOAC assay | No preoperative assay	Assay vs. No Assay: OR 2.98 (95% CI: 1.16–7.63), *p* = 0.023	NA	Major bleeding, Assay vs. No Assay: OR 1.44 (95% CI: 1–2.08), *p* = 0.050; Hb decrease 0.5066 g/L per 10 ng/mL DOAC titer (95% CI: 0.0299–0.9833, *p* = 0.037)	Major bleeding: Patients with a preoperative DOAC assay had a 1.44× higher odds of major bleeding (*p* = 0.05).	Reversal agent use, Assay vs. No assay: OR 16.3 (95% CI: 3.88–10.36), *p* = 0.0001; Embolization, Assay vs. No assay: OR 4.91 (95% CI: 1.09–22.11), *p* = 0.038; Median DOAC titer: 28 ng/mL [IQR 10–64, range <20–349 ng/mL); Median time from assay to surgery: 8.3 h (IQR 3.9–26.5); Time of last DOAC dose known in 42.2% of cases; 90.2% of DOAC assays ordered for surgeries with triage codes less than 24 h ago; Patients with DOAC assay older, with more emergent indications and undergoing higher risk operations
Godon, 2022 (N = 478) [49]	Registry data analysis	Urgent non-hemostatic invasive procedures in patients on Dabigatran (N = 160), Rivaroxaban (N = 274) or Apixaban (N = 44). 80% surgical procedures: 216/384 or 56% orthopedic, 75/384 or 20% GI surgery	None | NA	All-cause mortality: 28/478 (5.9%); 30-day mortality: 9/62 (15%) for patients with excessive bleeding and 19/416 (4.6%) for patients with no excessive bleeding (*p* = 0.005).	Major cerebral and cardiovascular events 7.9% (38/478) by day 30	Hemostatic agents administered in 16% (76/478) procedures. Excessive bleeding in 13% of procedures.	Excessive bleeding was observed in 13% of cases (62/478 procedures)	Hemostatic agents used in 16% of procedures, specific details not provided
Bradshaw, 2022 (N = 44) [50]	Single-center Retrospective	Emergent procedures in patients on Xa inhibitors. EVD 20.5%, laparotomy 13.6%, craniotomy 13.6%, Arterial embolization 20.5%, and others.	Andexanet alfa | NA	34.1% at 30 days	27.3% at 30 days (median 3.9 days to event)	Excellent/good hemostatic efficacy (38 patients): overall: 30/38 (78.9%); Intracranial: 12/15 (80%); Extracranial 18/23 (78.3%)	31.8% of patients required blood transfusions before andexanet alfa, and 27.2% required transfusions after reversal. Intra-procedure bleeding was more common in extracranial events but reduced after reversal.	PT decreased from 17.7 s to 16.8 s (*p* < 0.001); Anti-Xa activity decreased from 1.8 to 1.4 units/mL; Order to administration time: 30.0 min [IQR 19.8–43.0]; Door-to-reversal 2.6 h [IQR 1.2–5.5]; Reversal to procedure 2.8 h [ IQR 0.7–6.6]; LOS: ICU: 7 days [IQR 5.0–15.5]; Hospital: 11.5 days [IQR 6.0–19.0]
Levy, 2021 (N = 202) [51]	Multicenter prospective substudy	Urgent surgery/procedures in dabigatran-treated patients: Abdominal (49), orthopedic (45), vascular (34), neuro (8), Gynecological/urological (4)	Idarucizumab | NA	12.6% at 30 days, 18.9% at 90 days; Surgical group: 7.9%, Procedural group 7%	5% at 30 days, 7.4% at 90 days	93.4% normal, 5.1% mildly abnormal, 1.5% moderately abnormal	NA	Median time to procedure 1.6 h (3.3 h for neurosurgery); Symptom to treatment 292 min (IQR 195–419); Door-to-needle 136 min (IQR 92–181)
Shaw, 2020 (N = 82) [52]	Retrospective	DOAC-associated bleeding (65.9%) or preop optimization (34.1%). General, orthopedic, or interventional radiology.	aPCC | NA	30-day mortality: 31.7% or 26/82 patients; Fatal bleeding 17 or 20.7%	6.1% at 30 days	Normal hemostasis achieved (Preop cases only): 84% (21/25);	NA	INR: Pre-aPCC 1.6 [IQR 0.5] → Post-aPCC 1.2 [IQR 0.2], *p* < 0.00001; aPTT: Pre-aPCC 36 sec [IQR 12.8] → Post-aPCC 29 sec [IQR 9.8], *p* = 0.0001
Barzilai, 2020 (N = 62) [53]	Retrospective	Urgent surgery in apixaban/rivaroxaban patients; 61% abdominal surgery, 13% orthopedic, 10% cholecystostomy, 16% other procedures	PCC ± Tranexamic Acid | NA	21% at 30 days	3% (n = 2) (1 asymptomatic portal vein thrombosis post-ERCP, 1 superficial vein thrombosis from IV line 5 days post-PCC)	Hemostatic efficacy: 95% with no significant bleeding	Only 5% of patients (n = 3) experienced bleeding during surgery, and 26% (n = 16) received packed red blood cells (median: 1 unit, range: 1–5). No patients required additional PCC.	Drug levels measured in 11/62 patients (18%), mean drug level 116.5 ± 56 ng/mL; Median drug level 105 ng/mL; Tranexamic acid used in 14 patients in addition to PCC
Engelbart, 2018 (N = 42) [54]	Single-center case series	Life-threatening hemorrhage or urgent surgery: 71% ICH, 29% extracranial hemorrhage	FEIBA (all patients), ± TXA (52%)/vitamin K (5%)/FFP (5%)/Platelet transfusion (12%) | NA	29% overall (33% ICH, 17% extracranial)	10% (3 DVTs, 1 MI)	86% effective hemorrhage prevention	Hemorrhage progression occurred in 10% of patients (n = 4). Two patients were on apixaban, two on rivaroxaban, and some also on antiplatelet therapy (aspirin or clopidogrel).	FEIBA doses 25–50 units/kg, lower doses often effective
Emigh, 2021 (N = 606) [55]	Multicenter Prospective Observational	Trauma patients on DOACs with life-threatening hemorrhage or severe injury: 99% blunt trauma patients on DOACs requiring urgent procedure	Mixed reversal strategy (12% idarucizumab, 1% and exanet alfa, 80% 4-PCC, 7% FEIBA) | No reversal	11% vs. 3% (*p* = 0.001); 30% for specific vs. 8% for non-specific agents (*p* = 0.04); After multivariate analysis, reversal was not independently associated with mortality (*p* = 0.42)	No VTE complications reported	NA	NA	Injuries more severe in the reversal group: ISS [Injury Severity Score]: 16 vs. 5, *p* < 0.0001; Head AIS [Abbreviated Injury Scale]: 2.9 vs. 1.3, *p* < 0.0001; Abdominal AIS: 0.5 vs. 0.2, *p* = 0.05; LOS: Hospital: 8.9 vs. 4.6 days (*p* < 0.0001); ICU: 5.4 vs. 1.5 days (*p* < 0.0001)
